# Canadians' Dietary Intake from 2007 to 2011 and across Different Sociodemographic/Lifestyle Factors Using the Canadian Health Measures Survey Cycles 1 and 2

**DOI:** 10.1155/2019/2831969

**Published:** 2019-02-05

**Authors:** Zeinab Hosseini, Susan J. Whiting, Hassan Vatanparast

**Affiliations:** Division of Nutrition and Dietetics, College of Pharmacy and Nutrition, University of Saskatchewan, Saskatoon, SK S7N 2Z4, Canada

## Abstract

**Background:**

Nutrition is an important factor that impacts health, yet in Canada, there have been only a few surveys reflecting dietary intakes. The Canadian Health Measures Survey (CHMS) is a national survey that includes both food intake data as targeted questions and objective health measures. The aim of this research was to determine how food group intake data reported in CHMS is related to food group intakes from Canadian Community Health Survey (CCHS) (2004). A secondary objective was to examine the dietary status of Canadians across sociodemographic levels.

**Methods:**

The CHMS Cycles 1 and 2 food group intake data (meat and alternatives; milk products; grains; vegetables and fruits; dietary fat consumption; and beverages) of Canadians (6–79 years, *n*=11,387) were descriptively compared to previously reported intake of Canadians from CCHS 2.2 in 2004. Further, Canadians' food intakes were assessed across sociodemographic characteristics.

**Results:**

The CHMS dietary intake data from vegetables and fruits and from milk products groups were similar to the dietary intake reported from CCHS 2.2. For the other food groups, the difference in intakes suggested CHMS data by FFQ were not complete. However, similar patterns in food intakes with regards to age/sex and income were observed in both surveys.

**Conclusion:**

Not all food groups measured in CHMS provide complete dietary intake data as compared to CCHS 2.2, yet CHMS food group intakes provide valuable information when it comes to evaluating dietary intake across different population groups.

## 1. Introduction

Research has determined the impact of nutrition on health among populations; however, in Canada, only a few national surveys have included a dietary intake section. The first national nutrition survey, “Nutrition Canada” conducted from 1970 to 1972, indicated that Canadians had some nutrient inadequacies from diet and clinical or biochemical deficiencies (e.g., serum folate) [1]. The second comprehensive national survey was conducted in 2004 as a part of the series of Canadian Community Health Surveys (CCHS) [2]. This nutrition-focused survey was called CCHS 2.2 and included a repeated 24-hour dietary recall for collecting usual dietary intake data. These data showed low compliance of Canadians to the recommended intake of food groups [3].

The Canadian Health Measures Survey (CHMS) is a national survey that includes both food intake data and objective health measures. This survey is ongoing and has been running in biyearly cycles since 2007 [4]. Researchers have started using the dietary intake data from CHMS to determine the association between dietary intake and disease [5, 6]. The dietary intake data, which are derived from targeted food frequency questions, have yet to be evaluated in terms of whether they provide useful information on food intake to the health research community.

All dietary assessment methods have their strengths and limitations, which make them suitable for certain applications [7]. The food frequency questionnaire (FFQ) represents usual intake data of a period of time using a finite list of questions regarding usual intake data. This tool is less expensive, is more feasible, and has less random within-person variation than other methods; thus, it is a common candidate for large survey data [7]. However, this measurement tool is less accurate in estimating the nutrient composition of food and requires more cognitive task resulting in higher measurement error compared to a 24-hour dietary recall [8]. Although the latter tool is not as feasible as an FFQ, through its open-ended approach, more details regarding the variety and quantity of food are collected [7].

The aim of this research, therefore, is to determine how well the FFQ on CHMS encompasses current dietary guidance and represents the food consumption patterns of Canadians. To fulfill this purpose, results from CHMS are compared to previously published results form CCHS 2.2 [3]. In addition, through this research, we report food group intake data from the FFQ in CHMS combined Cycles 1 and 2 (2007–11). Further, the association of sociodemographics with dietary intake data is reported based on the CHMS data.

## 2. Subjects and Methods

### 2.1. Data Resource and Study Population

The CHMS is conducted by Statistics Canada in collaboration with Health Canada and the Public Health Agency of Canada [4]. Cycles 1 (2007–09) and 2 (2009–11) included approximately 5,600 (aged six to 79 years) and 6,400 (aged three to 79 years) participants, respectively, through a multistage sampling strategy [4, 9]. The survey covers almost 96.3% of the target population, which are all individuals living in Canada within the ages of three to 79 years. Excluded are people living in the territories; people living on reserve or in other Aboriginal settlements; fulltime members of the Canadian Forces; institutionalized residents; and people living in remote regions and regions with low population density [4, 9]. The adjusted final national response rate was 55.5% for Cycle 2 and 53.5% for the combined Cycles 1 and 2 [4, 9, 10]. The total number of respondents included in this study was 11,387 (6–79 y) for combined Cycles 1 and 2 data and 6,197 (4–79 y) for Cycle 2 data, which were representative of 29,625,300 and 30,680,029 of the Canadian population, respectively.

### 2.2. Dietary Assessment

The data from CHMS Cycles 1 and 2 can be combined using Statistics Canada's “Instructions for combining Cycle 1 and Cycle 2 data” document [11]. Thus, for this study, combined CHMS Cycles 1 and 2 data were used to compile the average daily consumption of food group intake (times/day) of Canadians (6–79 years) from targeted food frequency questions (indicated in Additional File 1). Included in this study are 32 questions in the CHMS FFQ grouped into four categories of meat consumption; milk and dairy product consumption; grains, fruits, and vegetables; dietary fat consumption; and water and soft drink consumption (water, salt and fish, and shellfish questions were excluded in this study) [10]. This dietary assessment tool has been developed with the aim of complementing physical and laboratory measurements. For example, the dietary fat-related questions in this tool are considered in the cardiovascular health research. However, this FFQ has not been validated. We named the food groups in this study based on Canada's Food Guide to Healthy Eating (1992) [12] to be comparable with CCHS 2.2 food groups assessed in 2004 used by Garriguet [3]. For the group of meat and alternatives, only CHMS Cycle 2 (4–79 y) data were used due to the inconsistency between fish and shellfish data from the two cycles of CHMS.

Previously, data from CCHS 2.2 collected using 24-hour dietary recalls were reported and compared to the Nutrition Canada Survey [3]. In the present study, results from CHMS dietary intake data, being the most recent dietary intake data available, were descriptively compared to the dietary intake data from CCHS 2.2 [3].

### 2.3. Dietary Intake by Sociodemographic Characteristics

Mean intakes by different sociodemographic and lifestyle characteristics were evaluated. The socioeconomic factors including age, sex, income, education, and physical activity were classified into their corresponding categories (e.g., two categories of male and female for sex) and by two age groups of 6–18 and 19–79 years. The age-sex specific categories were developed based on categories used by Garriguet [3], except for the first and last age categories, which were 6–8 y and 71–79 y for this study. Income was measured based on CHMS data of total household income, before taxes and deductions from all sources from everyone in the household since one year before the interview day, and considering the number of household members [13]. The lowest income category indicated < than $15,000 if one/two people; <than $20,000 if three/four people; and < than $30,000 if more than four people were living in the household. The lower-middle income category indicated having an income of $15,000–$29,999 if one/two people; $20,000–$39,999 if three/four people; and $30,000–$59,999 if more than four people were living in the household. The upper-middle category indicated income of $30,000–$59,999 if one/two people; $40,000–$79,999 if three/four people; and $60,000–$79,999 if more than four people were living in the household. Finally, the highest-level income indicated an income of $60,000 or more if one/two people and an income of $80,000 if more than two people were living in the household [13]. Education was categorized according to the CHMS household questionnaire classifications into four levels, only for 19- to 79-year-olds. Physical activity was indicated as the Physical Activity Index with the following categories in CHMS data set [13]: active, moderately active, and inactive.

For CCHS 2.2 (2004), Garriguet [3] characterised the age-sex groups based on the Institute of Medicine age categories for Dietary Reference Intakes [14]. The income variable categorization was similar to CHMS categorization [3, 13].

### 2.4. Data Analysis

Statistics Canada allows combining the first two cycles with the exception of a few variables that were not used in this research [13]. To ensure that data from the two consecutive CHMS Cycles 1 and 2 are representative, weighting and bootstrapping were applied and combining data from these cycles was done based on Statistics Canada's instructions [4, 11]. The intake of food groups/beverages was determined across demographic and socioeconomic factors as mean ± standard error. The mean estimate 95% confidence interval was used to evaluate significant differences between different levels. “No overlap” existing between CIs was considered as a statistically significant result [15]. IBM SPSS Statistics for Windows (v20, IBM Corp., Armonk, U.S.) was used for data processing, cleaning, and manipulation. STATA/SE (v11, StataCorp LP., College Station, U.S.) was used for statistical analysis.

## 3. Results

Based on the CHMS data sets used in this analysis Canadians reported having 1.62, 1.64, 4.33, 2.17, 0.47, 0.47, 0.14, and 0.7 serving/day from meat and alternatives; milk products; vegetables and fruits; grain products; dietary fat; sugar-sweetened beverages (SSB); diet drinks; and fruit and vegetable juice, respectively (Table 1).

**Figure 1:**
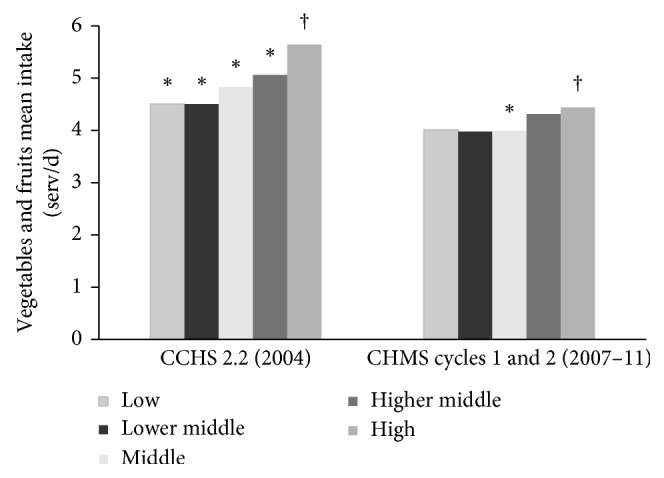
Canadian adults' vegetables and fruits mean intake (serving/d) differences by household income from the Canadian Health Measures Survey (CHMS) Cycles 1 and 2 and Canadian Community Health Survey (CCHS) 2.2 data from Garriguet (2007) [3]. ^*∗*^Significantly different from the reference group. ^†^Reference group.

Comparing the intake data of the four main food groups from CHMS with data reported from CCHS 2.2 [3], the total intakes from milk products and from vegetables and fruits were similar (Figure 1). Regarding the meat and alternatives and grain products intakes, fewer servings were reported in CHMS (1.62 and 2.17 servings/day, respectively, *p* < 0.05) compared to CCHS 2.2 (2.04–2.71 and 6.41–5.64 servings/day, respectively, *p* < 0.05 [3]) (Table 2).

Age differences in intake were apparent in CHMS (Table 2). Adults had significantly fewer serving intakes from SSB and fruit and vegetable juice (0.42 and 0.66 serving/day, respectively, *p* < 0.05) compared to children and adolescents (0.70 and 0.89 serving/day, respectively, *p* < 0.05). However, adults had more intakes from diet drinks (0.16 serving/day) compared to children and adolescents (0.05 serving/day, *p* < 0.05).

The dietary intake from CHMS among Canadians was different across sociodemographic characteristics including age, sex, education, income, and physical activity. Of all types of food groups examined, Canadian women (6–79 y) had more intakes from vegetables and fruits group and less from Grain Products and SSB group compared to Canadian men.

Based on CHMS 2007–11, within the age-sex groups, there were expected differences for Grain Products intake based on body size or energy requirements. For example, females in the 14–18 y age group have reported less intake from Grain Products (2.18 serving/day) compared to males in the same age group (2.54 serving/day, *p* < 0.05) or the males in the 19–30 y age group (2.42 serving/day) have reported more Grain Products intake compared to males in the 31–50 y age group (2.13 serving/day, *p* < 0.05).

### 3.1. Food Group Intake Based on Income and Education

Based on CHMS data from Cycles 1 and 2, the intake of Canadians were different across income and education levels for some food groups. Regarding income, within the 6–18 y and the 19–79 y age groups, the middle-income group (4.20 and 3.99 serving/day, respectively) had less intake from the vegetables and fruits group compared to the high-income group (4.60 and 4.44 serving/day, respectively, *p* < 0.05) (Figure 1). Similar results were observed in CCHS 2.2 [3]. Furthermore, according to data reported in CHMS, in the age range of 6–18 y, the low-and middle-income group had higher SSB intake (1.25 and 0.90 serving/day, respectively) compared to the high-income group (0.59 serving/day, *p* < 0.05) (Table 1).

Canadians aged 19–79 y, CHMS Cycles 1 and 2 data, with the less-than-secondary and secondary level of education had less intakes from the vegetables and fruits group (3.89 and 4.01 serving/day, respectively) and higher SSB intake (0.55 and 0.56 serving/day, respectively) compared to the group with postsecondary level of education (4.50 serving/day for vegetables and fruits intake and 0.32 serving/day for the SSB intake, *p* < 0.05).

### 3.2. Food Group Intake Based on Physical Activity

**Figure 2:**
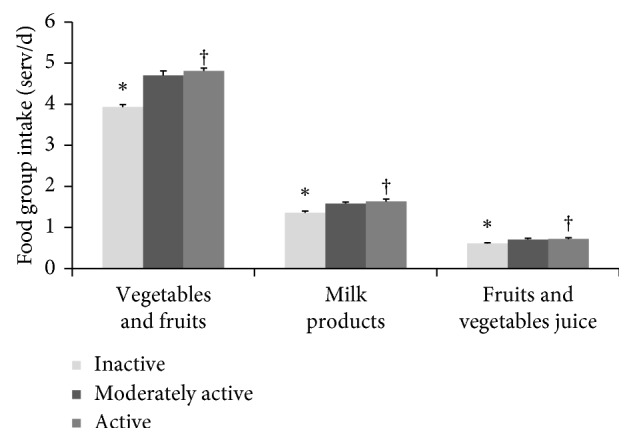
Canadian adults' (18–79 y) mean (±SE) dietary intake (serving/d) differences by level of physical activity. Canadian Health Measures Survey combined Cycles 1 and 2. ^*∗*^Significantly different from the reference group. ^†^Reference group.

The active group aged 6–18 y in CHMS 2007–11 had higher intake from the vegetables and fruits and milk products (4.44 and 2.37 serving/day, respectively) compared to the nonactive group in this age range (3.86 and 1.83 serving/day, respectively, *p* < 0.05), based on CHMS Cycles 1 and 2. Similarly, the active group aged 19–79 y had more intake from vegetables and fruits; milk products; and fruit and vegetable juice (4.81, 1.63, and 0.72 serving/day, respectively) compared to the inactive group (3.93, 1.36 and 0.61 serving/day, respectively, *p* < 0.05) within the same age group (Figure 2).

## 4. Discussion

This study extracted and reported dietary intake data from CHMS Cycles 1 and 2, a recent nationally representative health survey that included a dietary measurement. The present study provides evidence of the strengths and weaknesses of the FFQ as a dietary data collection instrument. These results demonstrate that, of all the types of food groups examined from CHMS data, Canadian women (6–79 years) had more intakes from vegetables and fruits and less from Grain Products and SSB compared to Canadian men. As well, the intake from the vegetables and fruits group varies across different sociodemographic and lifestyle factors including age, sex, education, income, and physical activity. Similar findings with regards to sex and income were observed in CCHS 2.2 by Garriguet [3].

We defined, for CHMS, the vegetables and fruits group based on Canada's Food Guide 1992 [12] in order to compare to CCHS. The impact of this food group in preventing chronic diseases such as cardiovascular diseases, diabetes, and cancer has been reported in several studies [16–18]. As a result, dietary guidelines have indicated that this food group is one of the essential food groups that should be consumed abundantly on a daily basis, and one, which should be monitored in the population. Based on the results of this study, Canadians' intake from the vegetables and fruits group reported in CHMS (2007–11) was similar to what was observed in CCHS 2.2 [3]. Moreover, the FFQ in CHMS has covered all items in the Health Canada vegetables and fruits group [19]. These agreements indicate that the CHMS FFQ vegetables and fruits food list is a complete list.

The CHMS FFQ did not include a few items from the milk products group indicated in Table 3. The analysis of this study also indicates Canadians' intake from milk products as reported in CHMS (2007–11) is similar to that from CCHS 2.2 [3]. This group is an important source of calcium, and therefore, accurate assessment of it is important from monitoring. However, a large difference was observed in between the intakes from the grains product group and from the meat and alternatives group between the two surveys. The intake from the grains group was much lower in CHMS (2007–11) compared to CCHS 2.2 [3]. This is probably due to not including the following grain products in the CHMS FFQ food list, which are among the commonly consumed grain products in Canada [19]: quinoa, bulgur, oatmeal, cornmeal, barley, buckwheat, rye, amaranth, millet, sorghum, triticale, couscous, pretzels, popcorn, crackers, pancakes, and waffles [19, 20]. Further, the CHMS FFQ does not cover intake from aggregated foods such as pizza, which are generally not easily captured by the FFQ method [21]. It is noteworthy that, in CHMS, whole-wheat and white grain products are not distinguished except for bread (e.g., brown rice vs. white rice) (Table 3).

Red meat, poultry, pulses and nuts, eggs, and fish have been the most consumed meat and alternatives products in Canada, respectively. However, the CHMS FFQ does not include questions regarding the intake from poultry, game birds, game meat, different beans, pulses, legumes, and foods such as hummus and tofu [10, 19]. In addition, other meats such as goat, rabbit, bison/buffalo, and veal are not included in the CHMS FFQ food list, which are not as common as the former foods listed [19] (Table 3). However, CHMS FFQ has covered red and processed meats; fish and shellfish; and egg intake, which are important food subgroups considered in investigating the association between different health outcomes and intake from these foods [22–25]. Based on the results of this study, the intake from the meat and alternatives group was lower in CHMS (2007–11) compared to Garriguet [3].

According to Statistics Canada, the initial aim of CHMS dietary intake data FFQ was to complement physical and lab measures. For example, the dietary fat intake questions are intended to provide useful information for the cardiovascular health panel, and the salt questionnaire is intended to complement the blood pressure panel. Thus, despite the targeted CHMS dietary intake questionnaire, this research indicates that questions from vegetables and fruits and the milk products intake mostly capture the intake from these food groups. However, the meat and alternatives and the Grain Products groups require the addition of a number of foods that have been indicated in Table 3, in order to represent intake from these food groups for the Canadian population.

Results from this study indicate that similar patterns were observed for the intake from different food groups between CHMS (2007–11) and CCHS [3]. Canadian women had more intakes from the vegetables and fruits group compared to men. Similarly, Garriguet [3] showed women of ages 19–30 years and 51 years and older had higher intakes from the vegetables and fruits group compared to males of the same age categories. As well, data from CCHS 2011 and 2012 using the vegetables and fruits FFQ indicated that Canadian women were more likely to have five or more servings per day intake from the vegetables and fruits group compared to males [3]. This difference observed between the sex is expected, given the higher nutritional knowledge among women compared to men [26].

Comparing the intake from the grains products across age-sex populations indicated patterns in energy intake in CHMS data. For example, overall males (2.23 serving/day) have had more intakes from the grains products compared to females (2.10 serving/day, *p* < 0.05), which was also observed in CCHS 2.2 [3]. As well, younger males aged 19–30 years (2.42 serving/day) have had more intakes from this food group compared to older males aged 31–50 years (2.13 serving/day, *p* < 0.05). Should grains be considered as a surrogate for energy intake, the pattern in energy intake across age-sex groups in CHMS (2007–11) was also observed in CCHS 2.2 (2004) [3]. Regarding milk products, based on CHMS dietary data, Canadians intake was progressively lower in younger age groups. Further, children and adolescents have had higher amounts of intake from milk products compared to adults, similar to the pattern observed in 2004 using CCHS 2.2.

Our study based on CHMS data reported the food group intake of Canadians by physical activity status; however, Garriguet [3] did not report intake by physical activity status. The CHMS data showed that active Canadians had more intakes from this food group compared to inactive Canadians. This is consistent with what was observed in a national study conducted in 2002, where active Canadians had higher intakes from the vegetables and fruits group compared to inactive Canadians [27]. They concluded that a higher intake from this food group is indicative of compliance to other healthy behaviours such as being physically active and nonsmoker [27]. The results from this research are supported by results from two other national studies, which indicate that sociodemographic and lifestyle factors impact the consumption rate of vegetables and fruits among Canadians [28, 29]. In addition, active Canadians have had higher intakes from Milk and Alternatives compared to inactive Canadians. This may be due to choosing an overall healthier lifestyle among this group of population [30].

The difference in the dietary intake observed in CCHS 2.2 (2004) and CHMS (2007–11) may be a result of factors including their difference in the dietary assessment tools used and the time interval between the surveys. The latter factor is dependent on whether the population of the two surveys is similar or not [31]. Both surveys are nationally representative, and there is only three years as an interval between the two surveys; therefore, it seems as if the population of the two surveys are rather comparable to each other. Therefore, this factor seems to have a minimal contribution to the difference observed between the reported data from the two surveys, while the former factor seems to be the main contributor to the difference observed between the food group intakes reported by the two surveys. The FFQ method is widely used in epidemiological studies due to its feasibility in terms of time, cost, and respondent burden [21]. However, this method has a higher measurement error compared to the 24-hour dietary recall method, which is used in CCHS [21]. Therefore, for using the FFQ method, it is recommended to investigate the dietary intake above the level of nutrients [21]. An additional point to consider is that in evaluating typical population dietary intakes, the day-to-day variation is an important factor. The FFQ method captures this variability at both the population and individual level [2, 21], whereas, a repeated 24-hour dietary recall considers this variation only at the population level after statistical adjustments [2, 21]. Therefore, considering the opportunity provided by CHMS to access objective nutrition-related health measures, the dietary intake for the vegetable and fruits and milk products reported by CHMS FFQ seems to be promising for investigating the diet-disease relationship not only at the population level but also at the individual level.

There are limitations of this study. The dietary intake data in CHMS were collected through a semiquantitative questionnaire, in which the frequency of intake was recorded rather than the quantity. In the analysis for CHMS, the youngest age-sex group included 6–8 year olds (missing ages 4 and 5 years) and the oldest group included 71- to 79-year-olds (missing ages above 79 years). The missing age groups are a small proportion of the overall age groups covered in CHMS. Moreover, the FFQ in CHMS does not cover all type of foods; therefore, this limitation should be considered while interpreting results. It may be possible for Statistics Canada to add the missing food items to the CHMS FFQ, as CHMS is an ongoing survey.

## 5. Conclusion and Implications

The CHMS is an ongoing Canadian survey that employs an FFQ for dietary assessment, which may cause confusion by health researchers as to the accuracy of dietary intake data. The reported meat and alternatives and Grain Products intake data should be used with caution as key foods in these groups were omitted from the FFQ. However, CHMS dietary intake data provide valuable information when it comes to evaluating the dietary intake across different population groups. Furthermore, a list of omitted foods from the CHMS FFQ has been presented in this study, which could be useful to both researchers and Statistics Canada.

## Figures and Tables

**Table 1:** Mean intake and 95% confidence interval of food intake (serving/day) of Canadians 6–79 y, by age-sex and income from the CHMS combined Cycles 1 and 2 (*n*=11,387, representative of population size: 29,625,300) (for meat and alternatives, only Cycle 2 was used; *n*=6,197, representative of population of 30,680,029).

	Meat and alternatives^1^ Mean (SE) (95% CI)	Milk products Mean (SE) (95% CI)	Vegetables and fruits Mean (SE) (95% CI)	Grains Mean (SE) (95% CI)
Total	1.62 (0.03) (1.55–1.7)	1.64 (0.03) (1.57–1.71)	4.33 (0.05) (4.23–4.42)	2.17 (0.02) (2.12–2.21)

Age-sex				
6–8 (M, F)	1.38 (0.033) (1.30–1.45)	3.01 (0.06) (2.88–3.14)	4.65 (0.10) (4.43–4.86)	2.50 (0.05) (2.40–2.60)
9–13 (M)	1.34 (0.05) (1.24–1.44)	2.68 (0.07) (2.53–2.83)^*∗*^'‡	4.37 (0.12) (4.13–4.61)	2.51 (0.05) (2.4–2.61)
9–13 (F)	1.30 (0.06) (1.17–1.42)	2.38 (0.06) (2.25–2.51)‡	4.70 (0.12) (4.44–4.95)	2.41 (0.05) (2.31–2.52)
14–18 (M)	1.51 (0.07) (1.36–1.66)	2.4 (0.1) (2.2–2.61)^*∗*^	3.99 (0.12) (3.76–4.23)	2.54 (0.07) (2.32–2.6)∗
14–18 (F)	1.33 (0.08) (1.17–1.50)	1.87 (0.09) (1.69–2.05)‡	4.38 (0.11) (4.15–4.61)	2.18 (0.06) (2.06–2.29)‡
19–30 (M)	1.92 (0.13) (1.65–2.20)^*∗*^	1.67 (0.11) (1.44–1.90)‡	4.08 (0.18) (3.71–4.45)	2.42 (0.08) (2.26–2.58)
19–30 (F)	1.44 (0.06) (1.30–1.58)	1.71 (0.07) (1.56–1.86)	4.30 (0.14) (4.02–4.58)	2.19 (0.05) (2.09–2.30)
31–50 (M)	1.77 (0.07) (1.61–1.92)	1.32 (0.04) (1.24–1.41)‡'^*∗*^	3.87 (0.08) (3.70–4.04)^*∗*^	2.13 (0.04) (2.04–2.22)‡
31–50 (F)	1.67 (0.09) (1.48–1.86)	1.55 (0.06) (1.42–1.68)	4.63 (0.10) (4.43–4.84)	2.06 (0.05) (1.95–2.17)
51–69 (M)	1.70 (0.05) (1.58–1.80)	1.20 (0.05) (1.11–1.29)^*∗*^	4.13 (0.10) (3.93–4.33)^*∗*^	2.07 (0.05) (1.97–2.18)
51–69 (F)	1.63 (0.07) (1.49–1.78)	1.50 (0.05) (1.40–1.61)	4.59 (0.11) (4.37–4.81)	1.96 (0.04) (1.87–2.04)
70–79 (M)	1.51 (0.05) (1.40–1.62)	1.45 (0.08) (1.28–1.62)	4.50 (0.09) (4.32–4.69)	2.19 (0.07) (2.05–2.33)
70–79 (F)	1.57 (0.08) (1.39–1.74)	1.62 (0.11) (1.39–1.84)	5.05(0.23) (4.57–5.53)‡	2.06 (0.09) (1.87–2.24)

Income (6–18 y)				
Low	1.20 (0.18) (0.82–1.59)	2.54 (0.19) (2.14–2.94)	4.63 (0.65) (3.30–5.97)	2.34 (0.32) (1.69–2.99)
Lower middle	1.41 (0.09) (1.21–1.61)	2.25 (0.15) (1.94–2.56)	4.12 (0.24) (3.62–4.62)	2.53 (0.07) (2.39–2.67)
Middle	1.47 (0.04) (1.39–1.56)	2.47 (0.08) (2.30–2.64)	4.20 (0.12) (3.94–4.45)^*∗*^	2.52 (0.05) (2.41–2.62)
Upper middle	1.36 (0.05) (1.25–1.48)	2.32 (0.06) (2.20–2.46)	4.25 (0.14) (3.97–4.53)	2.37 (0.05) (2.27–2.47)
High^†^	1.34 (0.05) (1.25–1.44)	2.55 (0.07) (2.41–2.69)	4.60 (0.07) (4.45–4.74)	2.38 (0.04) (2.31–2.46)

Income (19–79 y)				
Low	1.36 (0.15) (1.04–1.69)	1.49 (0.20) (1.08–1.90)	4.02 (0.34) (3.32–4.72)	2.09 (0.17) (1.74–2.44)
Lower middle	1.61 (0.09) (1.41–1.80)	1.49 (0.17) (1.14–1.84)	3.98 (0.25) (3.47–4.49)	2.39 (0.14) (2.10–2.67)
Middle	1.66 (0.11) (1.42–1.90)	1.44 (0.09) (1.27–1.62)	3.99 (0.12) (3.75–4.23)^*∗*^	2.20 (0.04) (2.11–2.29)
Upper middle	1.61 (0.06) (1.48–1.74)	1.46 (0.05) (1.37–1.55)	4.31 (0.08) (4.14–4.48)	2.12 (0.04) (2.03–2.18)
High^†^	1.74 (0.04) (1.66–1.83)	1.49 (0.03) (1.42–1.56)	4.44 (0.07) (4.28–4.59)	2.08 (0.03) (2.02–2.14)

CHMS: Canadian Health Measures Survey combined Cycles 1 and 2 (2007–11); CI: confidence interval; M: males; F: females; SE: standard error; y: years. ^1^For the Meat and Alternatives group, data from the Canadian Health Measures Survey Cycle 2 were used and ages 4–79 were included. ^*∗*^Significantly different from females of the same age group for the age-sex variable or from the reference group for income variable (*p* < 0.05). ^†^Reference group. ^‡^Significantly different from similar sex within the previous age group (*p* < 0.05).

**Table 2:** Canadians' food group intake reported from CHMS (2007–2011) and CCHS 2.2 data ([3]).

Food group	Children and adolescents Mean (SE^*∗*^) 95% CI		Adults ^†^ Mean (SE^1^) 95% CI	
	CHMS (2007–11, 6–18 y^‡^, *n*=4032)	CCHS^4^ (2004, 4–18 y)	CHMS (2007–11, 19–79 y, *n*=7355)	CCHS^§^ (2004, above 18 y)
Meat and alternatives	1.37 (0.03) (1.32–1.43)	2.04 (1.99–2.09)	1.68 (0.04) (1.60–1.77)	2.71 (2.64–2.76)
Milk products	2.46 (0.05) (2.37–2.55)	2.29 (2.24–2.35)	1.47 (0.03) (1.40–1.54)	1.52 (1.48–1.56)
Vegetables and fruits	4.41 (0.06) (4.29–4.53)	4.45 (4.34–4.56)	4.33 (0.05) (4.23–4.42)	5.16 (5.05–5.26)
Grain products	2.41 (0.03) (2.35–2.47)	6.41 (6.30–6.53)	2.12 (0.03) (2.07–2.17)	5.64 (5.53–5.75)

CCHS: Canadian Community Health Survey; CHMS: Canadian Health Measures Survey combined Cycles 1 and 2 data; CI: confidence interval; *n*: sample size; SE : standard error; y: years.^*∗*^The report by Garriguet [3] did not provide standard error values. ^†^Adults age range for CHMS is 19 to 79, and it is 19 and above for CCHS 2.2. ^‡^Children and adolescents age range for CHMS is 6–18 y for all food groups except for meat and alternatives, which is 4–18 y. The age range of children and adolescents for CCHS 2.2 is 4–18 y for all food groups. ^§^Data from Garriguet [3].

**Table 3:** The list of missing foods in the CHMS Cycles 1 and 2 nutrition questionnaire (based on intake of food in Canada and Health Canada food list) [19].

Food groups^*∗*^	Missing foods in the CHMS dietary questionnaire
Meat and alternatives	(i) Pulses/legumes (except dried beans) and some foods made of them such as hummus and tofu (ii) Poultry (such as chicken, turkey, and duck) and game birds (iii) Game meat (such as deer, moose, caribou, and elk) (iv) Other meats such as deli meat, goat, rabbit, bison/buffalo, and veal.

Milk products	(i) Block/processed cheese (such as cheddar, mozzarella, Swiss, and feta) (ii) Other types of cheese such as cottage, quark, goat cheese, and paneer (iii) Buttermilk (iv) Kefir (v) Pudding/custard (vi) Yoghurt drink

Grains products	(i) Brown pasta and white pasta (ii) Brown rice and white rice (iii) Crackers, popcorn, pancakes, pretzels, and waffles (iv) Oatmeal, quinoa, bulgur, couscous, cornmeal, buckwheat, barley, rye, amaranth, millet, sorghum, triticale, or any other grains (v) Aggregated foods such as pizza

Vegetables and fruits	—

Dietary fat	(i) Butter (ii) Margarine (iii) Unsaturated vegetable oils such as canola, corn, flaxseed, olive, peanut, soybean, and sunflower

Beverages	(i) Coffee (ii) Tea

^*∗*^Based on the 1992 Canada's Food Guide [12] and reported by Garriguet [3].

## Data Availability

Data from Canadian Health Measures Survey are confidential and protected. Such data are only available at Statistics Canada Research Data Centers. Any results can be vetted and published.

## Supplementary Materials

Supplementary Materials Table 1. Grouping of CHMS dietary intake questions.
