# Supplementary material for: Canadians' Dietary Intake from 2007 to 2011 and across Different Sociodemographic/Lifestyle Factors Using the Canadian Health Measures Survey Cycles 1 and 2
Source: J Nutr Metab. 2019 Feb 5;2019:2831969. doi: 10.1155/2019/2831969 (PMC6379838; doi:10.1155/2019/2831969)
Supplement: Supplementary Materials — Table 1. Grouping of CHMS dietary intake questions. [file 2831969.f1.docx]

**Supplement File 1**

**Table 1**. Grouping of CHMS dietary intake questions.

| Food groups^1^ | i.e. “how often do you usually eat per day/week/month/year?”, otherwise the whole question is asked^2, 3^. |
| --- | --- |
| CHMS Questions on Meat and Alternatives group | -Beef or pork hot dogs  -Cooked dried beans, such as refried beans, baked beans, pea soup or kidney beans, excluding green and yellow beans  -Eggs and egg dishes including the yolk (excluding all egg dishes made with only egg whites); egg dishes could include eggs, omelettes, frittata or quiche.  -Fish- and shellfish-related questions in Cycle 2 [3]  -Liver (including all types of liver such as beef, veal, pork or chicken, but excluding liverwurst and liver pâté)  -Other organ meats such as kidneys, heart or giblets  -Peanuts, walnuts, seeds, or other nuts, excluding nut butters such as peanut butter  -Red meat (beef, hamburger, pork or lamb)  -Sausage or bacon (including all types of sausage, such as breakfast, pepperoni and kielbasa but excluding low-fat, light or turkey varieties) |
| CHMS questions on Milk and Alternatives group | -Cottage cheese  -Milk or enriched milk substitutes. Questions are asked about the kinds of milk usually consumed (3.25, 1, 0.5, skim or non-fat), flavoured milk beverages (chocolate milk and flavoured milk beverages such as Oh Henry®, rice, soya and other).  -Yogurt, excluding frozen yogurt  -Ice cream or frozen yogurt |
| CHMS questions on Grain products group | -Any kind of pasta (including spaghetti, noodles, macaroni & cheese or pasta salad)  -Any kind of rice  -Brown bread, including bagels, rolls, pita bread or tortillas  -Hot or cold cereal  -Instant, seasoned or wild rice (such as Minute Rice®, Dainty®, Rice-a-Roni®)  -White bread, including bagels, rolls, pita bread or tortillas |
| CHMS questions on Vegetables and Fruit group | -Fruit (fresh, frozen or canned)  -Lettuce or green leafy salad with or without other vegetables  -Other than French fries, home fries, or hash brown potatoes, including baked, boiled, mashed or in potato salad, but excluding sweet potatoes  -Spinach, mustard greens or collards excluding kale  -Tomatoes or tomato sauce, including salsa, tomato soup and spaghetti sauce but excluding tomato paste, ketchup or pizza sauce  -French fries, home fries, or hash brown potatoes  -Fruit and vegetables juice (includes fruit juice and vegetables juice)  -All other types of vegetables excluding those already mentioned |
| CHMS questions on dietary fat | -Regular-fat potato chips, tortilla chips or corn chips (excluding low fat chips and pretzels).  -Regular-fat salad dressing or mayonnaise (including on salads and sandwiches) |
| CHMS questions on beverages | -Diet soft drinks  -Sugar sweetened beverages (includes fruit flavoured drinks, regular soft drinks, sport drinks, such as Gatorade® or PowerAde®)  -Fruit and vegetables juice (includes fruit juice and vegetables juice) |

^1^ Food groups are named based on the 1992 Canada’s Food Guide and reported in Garriguet^3^.

**Footnotes:**

1. Garriguet D. Canadians' eating habits. Health Rep. 2007;18(2):17.
2. Statistics Canada. Canadian Health Measures Survey – Data Dictionary: Cycle 1. Ottawa. ; 2010, [cited 2015 Oct 20] Available at <http://www.library.carleton.ca/sites/default/files/find/data/surveys/pdf_files/chms-c1-07-09-dic.pdf>
3. Statistics Canada. Canadian Health Measures Survey (CHMS) Data User Guide: Cycle 2, Ottawa; 2012 [cited 2015 Oct 18], Available at <http://data.library.utoronto.ca/datapub/codebooks/cstdli/chms/CHMS_User_Guide_Cycle2_E.pdf>
